# The lung microbiome in children with HIV-bronchiectasis: a cross-sectional pilot study

**DOI:** 10.1186/s12890-018-0632-6

**Published:** 2018-05-22

**Authors:** Refiloe Masekela, Solize Vosloo, Stephanus N. Venter, Wilhelm Z. de Beer, Robin J. Green

**Affiliations:** 1 Department of Paediatrics and Child Health, Faculty of Health Sciences, University of Pretoria, Pretoria, South Africa; 2 Department of Maternal and Child Health, Nelson R Mandela School of Medicine, College of Health Sciences, University of KwaZulu-Natal, 719 Umbilo Road, Congella, Durban, 4013 South Africa; 3 Department of Microbiology and Plant Pathology, University of Pretoria, Pretoria, South Africa

**Keywords:** Paediatrics, Microbiology, HIV-associated bronchiectasis, Bacterial diversity, Lung microbiome

## Abstract

**Background:**

Data on the lung microbiome in HIV-infected children is limited. The current study sought to determine the lung microbiome in HIV-associated bronchiectasis and to assess its association with pulmonary exacerbations.

**Methods:**

A cross-sectional pilot study of 22 children (68% male; mean age 10.8 years) with HIV-associated bronchiectasis and a control group of 5 children with cystic fibrosis (CF). Thirty-one samples were collected, with 11 during exacerbations. Sputum samples were processed with 16S rRNA pyrosequencing.

**Results:**

The average number of operational taxonomy units (OTUs) was 298 ± 67 vs. 434 ± 90, for HIV-bronchiectasis and CF, respectively. The relative abundance of *Proteobacteria* was higher in HIV-bronchiectasis (72.3%), with only 22.2% *Firmicutes*. There was no correlation between lung functions (FEV_1_% and FEF_25/75_%) and bacterial community (*r* = 0.154; *p* = 0.470 and *r* = 0.178; *p* = 0.403), respectively. Bacterial assemblage of exacerbation and non-exacerbation samples in HIV-bronchiectasis was not significantly different (ANOSIM, R_HIV-bronchiectasis_ = 0.08; *p* = 0.14 and R_CF_ = 0.08, *p* = 0.50). Higher within-community heterogeneity and lower evenness was associated with CF (Shannon-Weiner (H′) = 5.39 ± 0.38 and Pielou’s evenness (J) 0.79 ± 0.10 vs. HIV-bronchiectasis (Shannon-Weiner (H′) = 4.45 ± 0.49 and Pielou’s (J) 0.89 ± 0.03.

**Conclusion:**

The microbiome in children with HIV-associated bronchiectasis seems to be less rich, diverse and heterogeneous with predominance of *Proteobacteria* when compared to cystic fibrosis.

## Background

Bronchiectasis is a chronic inflammatory lung disease that, in high-income countries, has been declining outside of the context of cystic fibrosis (CF) in children, as compared to adults where the incidence and prevalence is on the rise [1]. However, this is not so in low-middle income countries and some economically disadvantaged groups in high-income countries [2–4]. The burden of disease is linked to inequity in access to quality health care, lack of essential medicines, high tuberculosis (TB) rates, indoor pollution and secondary immunodeficiency states such as human immunodeficiency virus (HIV) infection [5].

Bronchiectasis is characterized by interspersed episodes of quiescence and pulmonary exacerbations (PEs). The consequence of PEs is chronic respiratory disability and poor quality of life. A key factor in the initiation of PEs are airway microbes, which are thought to establish recurrent respiratory tract infections and therefore maintain an inflammatory milieu in the airway [6]. Traditionally, microorganisms are obtained from respiratory samples via microscopy and culture and this is then utilized to guide anti-microbial therapy. Recently, there has been a renewed interest in research on the microbial community in the lung of individuals in both diseased and in healthy lungs; this research is based on culture-independent phylogenetic profiling approaches based on genetic biomarkers such as 16S rRNA sequencing [7, 8].

Microbial communities isolated in the upper airways have been found to closely resemble those present in the lung compartment [9]. In the context of both CF and non-CF bronchiectasis, there is evidence that bacterial diversity is critical in the maintenance of “homeostasis” and that this prevents PEs and is associated with better lung function [10, 11]. The contribution of microbes to the specific community of individuals’ lungs may either suppress (resilience microbiota) or precipitate (risk microbiota) pulmonary exacerbations [8, 10]. In the context of HIV infection, which is known to affect both innate and adaptive immune pulmonary responses, recent data suggests that there is a change in the lung microbiome of HIV-infected individuals which is attributed to the immunosuppressive state; however these studies have been in adult cohorts [12, 13].

To our knowledge, there is currently no published data on the airway microbiome in children with HIV-associated bronchiectasis on antiretroviral therapy and the changes in the microbiome during or between exacerbations episodes. The primary aim of this pilot study was to evaluate the microbiome in HIV-infected children with established chronic lung disease, to assess the diversity of the microbiome, and to assess for any changes that may occur during exacerbation episodes. We also sampled a small group of children with bronchiectasis secondary to cystic fibrosis to evaluate any differences between these children and those with HIV-bronchiectasis in the same environment.

## Methods

### Setting

Children were recruited during routine or unscheduled visits at the Steve Biko Academic Hospital, Chest Clinic, Pretoria, South Africa during a 17-month period between May 2013 and October 2014. This clinic serves as a referral centre for children from Tshwane Metropolitan region in Gauteng with over 2.5 million children living in a peri-urban setting, where 27.0% of the people live in informal settlements with a high HIV prevalence rate at 11.2% in 2015. The clinic also serves the adjoining Mpumalanga province with a largely rural population. All the children in the study were from communities in Tshwane (urban/pre-urban) and Mpumalanga province (rural). For the HIV-bronchiectasis group, HIV status was based on a positive enzyme-linked immunosorbent assay (ELISA). All subjects had to have been on antiretroviral therapy for a minimum of 6 months prior to enrolment. All children at the clinic are screened routinely every 3 months for TB and none of the subjects had positive TB cultures. Subjects with cystic fibrosis (CF) confirmed by genetics and/or two positive sweat tests were invited to participate to serve as controls in a 3:1 design.

Exacerbations were defined according to the following criteria: a change in the nature of cough or increasing shortness of breath; development of new constitutional symptoms (fever, malaise) or changes in sputum characteristics (e.g. sputum colour and/or increase in sputum quality and/or increase in sputum volume). The sputum quality was assessed using the Barlett score which is based on the average number of neutrophils per low power field, average number of epithelial cells per low power field and presence of mucus/saliva in the specimen [14]. A value of < 0 indicates either either no inflammation or a poor quality specimen. Immune staging with HIV viral load and CD4^+^ T cells was performed. Presence of bronchiectasis was confirmed for each child by a CT chest scan carried out by an independent radiologist and pulmonologist. Lung function testing was performed using Viasys SpiroPro Jaeger Spirometer (Hoechberg, Germany).

### Sputum collection, processing and DNA extraction

All sputum samples were collected by sputum induction after nebulization with hypertonic saline and collected by mucus extractors after percussions by a physiotherapist. Prior to DNA extraction, the sputum samples were washed with two times the volume, 0.85% Phosphate Buffered Saline (PBS) (8.00 g/L NaCl, 0.2 g/L KCl, 1.44 g/L Na_2_HPO_4_, 0.24 g/L KH_2_PO_4_, pH 7.4). Excess PBS was removed and the remaining sputum was incubated with equal volume Sputasol (Thermo Scientific), a mucolytic agent, at 37 °C. The liquefied suspension was centrifuged at 11000 x g for 5 min. The supernatant was removed and the pellet was washed with 750 μl PBS and centrifuged at 10000 x g for 5 min. The wash step was repeated two more times. DNA was extracted from the processed sputum samples using the Zymo Research Genomic DNA™ Tissue MiniPrep kit (Zymo Research, South Africa), in accordance with the manufacturer’s protocol. The protocol includes a pre-treatment step with Proteinase K to improve lysis of Gram-positive bacteria. DNA concentration and purity (OD260/280 and OD 230/260) were determined using the Nanodrop ND-1000™ Spectrophotometer. All genomic DNA was stored at − 20 °C until further analysis.

### 16S rRNA gene amplification and pyrosequencing

Full length 16S rRNA libraries were constructed using primers: 27F (5′AGAGTTTGATCCTGGCTCAG-3′) and 1492R (5’-GGTTACCTTGTTACGACT-3′) adapted from Edward et al. [15]. In order to increase sequencing depth, five 16S rRNA amplicon libraries were constructed for each sample. The five generated amplicon libraries of corresponding samples were pulled and sent to Inqaba Biotec (Pretoria, South Africa) for variable region, V1-V3 amplicon library construction and pyrosequencing, using the GS Junior System (Roche Applied Science, Basel). Bacterial 16S rRNA amplicons obtained were subjected to variable region V1 – V3 bacterial community profiling, using the 454-pyrosequencing platform. Polymerase chain reaction (PCR) was carried out using the BIO-RAD T100™ Thermal Cycler. The polymerase chain reaction (PCR) mixtures (25 μl) consisted of 1 x reaction buffer, 1.5 mM MgCl_2_, 250 μM of each nucleotide (dATP, dCTP, dGTP, dTTP), 10 pmol of each primer (forward and reverse), 1.5 U Taq DNA polymerase, 16.85 μl nuclease free water (Qiagen) and 0.5 μl genomic DNA. The cycling conditions for the 16S rRNA amplicons consisted of an initial denaturation step at 92 °C for 10 min, followed by 30 cycles of denaturation at 92 °C for 1 min, annealing at 58 °C for 1 min, extension at 75 °C for 1 min, and a final extension at 75 °C for 5 min. At the end of the 30 cycles, the reaction was kept at 4 °C. Each DNA amplification step within the 16S profiling process included standard negative controls using nuclease-free water instead of the sample DNA. Samples were not processed to the next step unless the negative controls were confirmed to be negative. During the study, all negative controls showed no amplification. Standard negative controls were also included during the sequencing process. No extra measures typically required for low microbial biomass samples were performed, as all the DNA extractions yielded high concentrations of DNA (determined with nanodrop) and no more than 30 cycles were required for any of the PCR amplification steps [16]. Normal positive control samples (bacterial DNA) as well as the positive reactions obtained for all the samples indicated that the various steps in the analysis provided the expected results.

### Sequence processing and data analysis

Sequence processing and data analysis were conducted using the MOTHUR software package (version 1.35.1) and processing pipeline as described on the MOTHUR website: www.mothur.org/wiki/454_SOP [17]. Briefly described, raw pyrosequencing reads were initially screened to remove all sequences that did not meet the required quality criteria. Processed sequences following initial screening included sequences with a minimum quality score of 35, minimum sequence length of 150 nucleotides, maximum sequence length of 600 nucleotides, maximum of six ambiguous nucleotides and absence of mismatches in barcodes and primers. Following quality filtering the processed sequences were aligned to a reference alignment, which was generated from the SILVA seed ribosomal RNA database (Release 119) [18]. After alignment, the sequence data set were screened to cull all sequences that did not align to the alignment region, variable region V1 – V3 of the 16S rRNA gene. The ends of the aligned sequences were subsequently trimmed to ensure that the sequences all started and ended at the same alignment coordinates. The aligned sequences were screened for chimeras using UCHIME [19]. Taxonomic affiliation was assigned to each processed and chimeric-free sequence using the GreenGenes reference taxonomy database at a pseudobootstrap confidence score of 80%. Unwanted lineages were culled by removing sequences that could not be classified to kingdom level, or that classified as *Eukaryota*, chloroplast, or mitochondria. The remaining high quality reads were clustered into operational taxonomic units (OTUs) at a 97% similarity threshold. Representative sequences for each OTU were obtained and classified against the GreenGenes dataset (August 2013 of gg_13_8) as described above.

### Sample diversity comparisons and statistics

In order to ensure that all samples were compared at the same sequence depth, a computation of alpha and beta diversity indices was performed. This was performed after sub-sampling of the entire sequence dataset 1000 times to a defined number of sequences. The sub-sampling threshold was determined following rarefaction analysis. The rarefaction curves of the samples reached completed saturation at about 1200 sequences per sample. In light of this, the sequence database were subsampled to a threshold of 1200 sequences per sample. Alpha and beta diversity indexes were calculated using functions provided in the MOTHUR software package (version 1.35.1) [17]. Three alpha diversity indexes, e.g. Chao1, Shannon-Weiner index (H′) and Pielou’s evenness index (J) were calculated. Chao1 was used as a measure of within community species richness, whereas H′ and J were used as measures of within community heterogeneity and evenness, respectively.

For OTU-based beta diversity analysis, variability in the bacterial species assemblage between samples was analysed using two ecological coefficients of compositional dissimilarity, namely, Jaccard and Bray-Curtis [20, 21]. Jaccard coefficients were used to address community structure, as calculated pair-wise dissimilarity among selected samples is based on incidence-data (presence/absence), whereas Bray-Curtis coefficients were used to address community membership, as pair-wise dissimilarity between selected samples is calculated on the basis of incidence and abundance data. In addition, comparative analysis of compositional variability within the community assemblage of individual samples was visualized by performing non-metric multi-dimensional scaling (NMDS) on the Bray-Curtis distances using the vegan package (metaMDS function) in R [22]. This was followed by the analysis of similarities (ANOSIM) to statistically explain the compositional variability observed among samples categorized according to defined groupings [23].

Analysis included descriptive statistics for age, gender and lung functions. Associations between bacterial communities, disease, exacerbations and lung function parameters i.e. FEV_1_% predicted and FEF_25-75_% were investigated using Pearson correlation coefficients utilizing STATA 13.0 **(**StataCorp LP. 2013 Stata: Release 13, College Station, TX, USA). For all statistical analyses, the null hypothesis was rejected at a probability of *p* < 0.05. Written informed consent was provided by parents or guardians for all children under the age of 18 years and assent for all children over the age of 7 years. Ethical approval for the study was granted by the Research Ethics Committee of the Faculty of Health Sciences of the University of Pretoria (HREC No 315/2013).

## Results

### Clinical data

The demographics and baseline data of the 27 subjects recruited for the study are reflected in Table 1. The 22 HIV-bronchiectasis subjects (72% male) included had a mean age of 10.8 years. For the CF controls, six patients were enrolled; of these, one patient was excluded from analysis due to poor sputum quality. The final analysis therefore included only 5 subjects (60% males) with a mean age of 8.4 years.

**Table 1:** Demographic, immunological and lung function data of children with HIV-associated bronchiectasis and CF-bronchiectasis

Variable	HIV associated bronchiectasis		CF-bronchiectasis ^δ^	
	Mean	95% CI	Mean	95% CI
Age (years)	10.8	9.4–12.3	8.4	6.9–9.7
Gender (M/F)*	16/6 (72/28)		3/2 (60/40)	
Height z score **	−2.3	−2.9 – −1.46	−1.0	−4.3 – 2.2
BMI z-scores**	−1.9	−2.9 – −0.6	−0.9	−3.5 – 1.6
CD4% count	22.9	19.3–25.5		
HIV-viral load (copies/ml)	11,455	1768–74,199		
Duration HAART (months)	48.0	34.5–62.6		
FEV_1_% predicted	52.5	45.6–59.4	84.8	45.5 –124.0
FEF _25/75_% predicted	47.8	36.6–59.1	72.7	63.2–82.3
Bartlett score^§^	1.6	1.4–1.9	1.75	0.9–2.5
Mutation (%)			p.F508del (67)	3120 (33)

*numbers expressed in parentheses percentage of males and females; **: height and body mass index expressed as z-scores (SD) as per WHO criteria with normal between 0 and 2 z-scores; ^§^ Bartlett score from reference 14; ^¶^p.508.del^:^ p^.^F508del./p.F508.del; 3120: 3120 = 1G > A/3120 + 1G > A; ^**δ**^ results for 5 children

In total, 31 sputum samples were collected. Twenty-one sputum samples (HIV-bronchiectasis = 18 and CF = 3) were collected from clinically stable subjects (non-exacerbation samples); the remaining ten samples were collected prior to the initiation of antibiotics for an exacerbation (HIV-bronchiectasis = 8 and CF = 2).

The HIV-bronchiectasis subjects had been on highly active antiretroviral therapy (HAART) for a mean duration of 4 years, and WHO stage 4 with evidence of moderate immune suppression and inadequate HIV viral suppression. Of these children, 4 had HIV-viral loads greater than 100,000 copies/ml, despite being on HAART for more than 6 months reflecting treatment failure. The respiratory morbidity in the HIV-bronchiectasis group was severe with a lower mean FEV_1_% predicted and significant lower airway obstruction. For the CF group, the children were younger with more preserved lung function when compared to the HIV-bronchiectasis group.

### Pyrosequencing data analysis

The total number of raw 16S rRNA variable region, V1 – V3 pyrosequencing reads were 223,458, with a Mean ± *SD* of 6983 ± 12,146 per sample. The average number V1 – V3 pyrosequencing reads of processed sequences obtained within HIV-bronchiectasis and CF samples were (mean ± SD) 3762 ± 2568 and 1409 ± 283, respectively. Subsequent classification of the processed sequences into operational taxonomic units (OTUs) at a 97% similarity threshold identified 4779 OTUs. The average number of OTUs detected among HIV-bronchiectasis samples were (mean ± SD) 298 ± 67, whereas those for CF samples were 434 ± 90.

### Bacterial diversity analysis

**Fig. 1:**
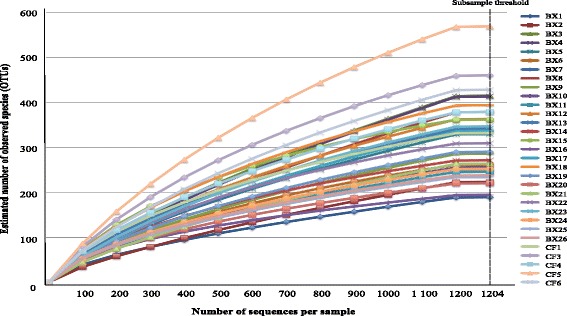
Rarefaction analysis displaying estimated number of observed species (OTUs at 97% similarity) detected at different sequence intervals. The subsampling threshold limit was set at 1204 sequences per sample (dotted black line). BX: bronchiectasis and CF: cystic fibrosis

**Fig. 2:**
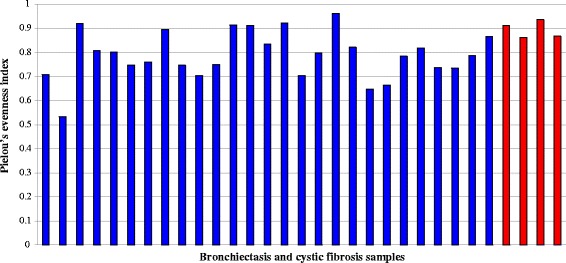
Pielou’s evenness indexes calculated for individual bronchiectasis (blue) and cystic fibrosis (red) samples

**Fig. 3:**
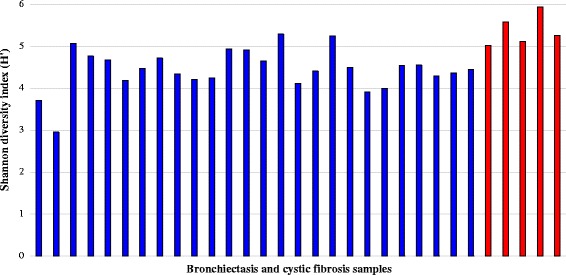
Shannon diversity indexes (H′) calculated for individual bronchiectasis (BE, blue) and cystic fibrosis (CF, red) samples

The visual display of the rarefaction curves infers a continued emergence of new observed species as the sequence output increases (Fig. 1). The rarefaction curves of the samples reached completed saturation at about 1200 sequences per sample. In light of this, the sequence database was subsampled to a threshold of 1200 sequences per sample. Following computation Chao1, Shannon-Weiner (H′) and Pielou’s evenness indices (J), there were no significant differences in Chao1 [F(1, 29) = 0.69, *p* = 4.12E-01); however, there was significant differences in Shannon-Weiner (H) [F(1, 29) = 16.22, *p* = 3.72E-04] and Pielou’s (J) [F(1, 29) = 5.26, *p* = 3.00E-02]. Specifically, the community of the CF samples was significantly more diverse (H′, mean ± SD = 5.39 ± 0.38) and uneven (J, mean ± SD = 0.79 ± 0.10) when compared with the HIV-bronchiectasis samples (mean ± SD for Shannon-Weiner = 4.45 ± 0.49 and Pielou’s 0.89 ± 0.03, respectively) (Figs. 2 and 3).

**Fig. 4:**
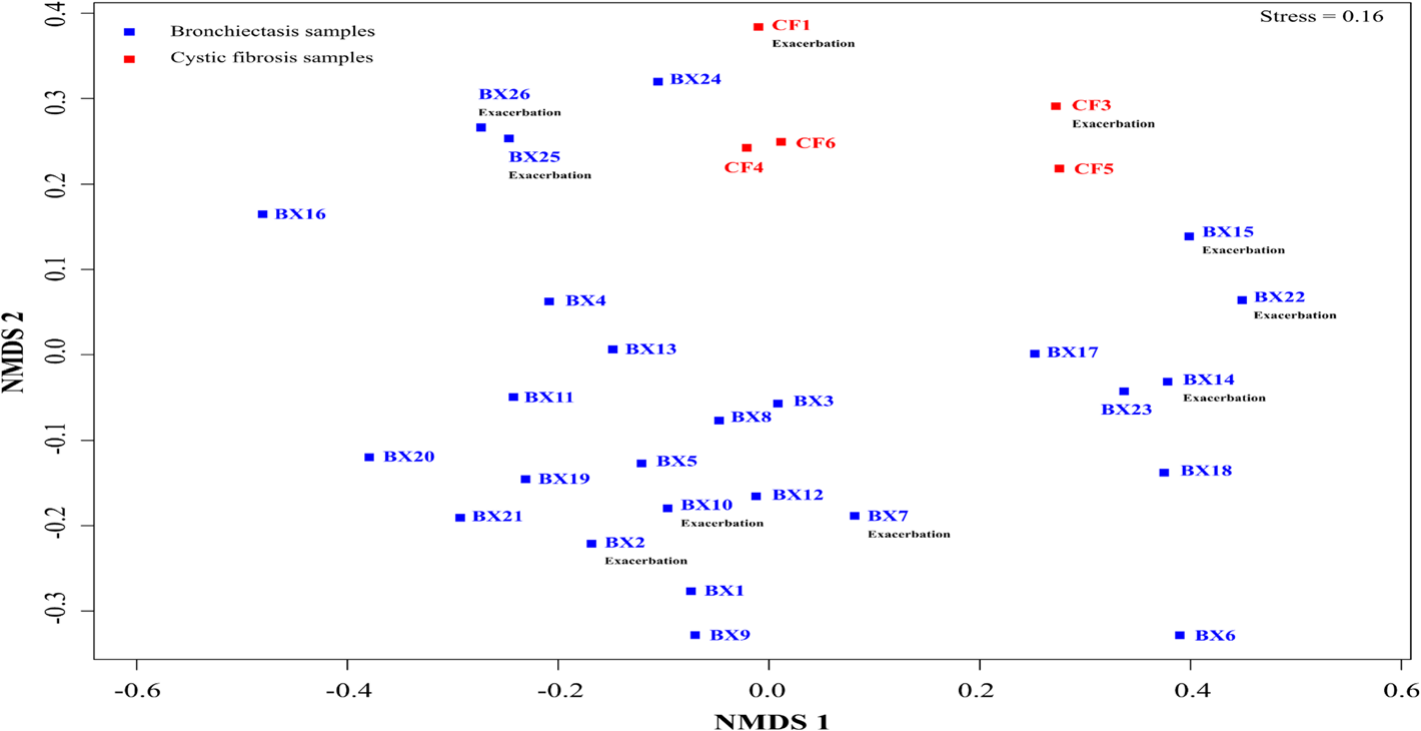
Two-dimensional nonmetric multidimensional scaling (NMDS) plot displaying the spatial ordination of 31 sputum samples collected from 22 bronchiectasis subjects (BX, blue) and 5 cystic fibrosis subjects (CF, red)

Jaccard (D_J_) and Bray-Curtis (D_BC_) were used to compare the bacterial community structure and membership between samples. Within the HIV-bronchiectasis group, the average dissimilarity in the community membership was about 92% (D_J_, mean ± SD = 0.92 ± 0.08), whereas the average dissimilarity in the community structure was about 95% (D_BC_, mean ± SD = 0.95 ± 0.07). Similarly, within the CF group the average dissimilarity in the community membership was about 80% (D_J_, mean ± SD = 0.80 ± 0.10), whereas the average dissimilarity in the community structure was about 88% (D_BC_, mean ± SD = 0.88 ± 0.05). To depict the degree of compositional variability amongst the HIV-bronchiectasis and CF samples, all the samples were ordinated in a two-dimensional nonmetric multidimensional scaling (NMDS) plot (based on Bray-Curtis dissimilarity measures) (Fig. 4). To test for localized bacterial community assemblage confined to HIV-bronchiectasis and CF groups, analysis of similarity test (ANOSIM) test was performed using on Bray-Curtis distances. Following ANOSIM tests there was a significant difference in the community structures of the HIV-bronchiectasis and CF samples (ANOSIM, *R* = 0.21, *p* = 0.04). In contrast, there was no significant difference in the community structures between the exacerbation and non-exacerbation samples for either disease groups (ANOSIM, R_HIV-bronchiectasis_ = 0.08, *p* = 0.14; R_CF_ = 0.08, *p* = 0.50).

### Bacterial community profiling

For bacterial community profiles, eight bacterial phyla – *Actinobacteria*, *Bacteroidetes*, *Firmicutes*, *Fusobacteria*, *Proteobacteria*, *Spirochetes*, *Tenericutes* and *Candidatus Saccharibacteria* were recovered from all samples. Three phyla – *Spirochetes*, *Tenericutes* and *Candidatus Saccharibacteria* were encountered only within the bronchiectasis group, where they were infrequently detected at relative abundances < 1%. *Proteobacteria* and *Firmicutes* were the two dominating phyla detected within the HIV-bronchiectasis and CF groups with combined average relative abundances of these two phyla reaching 94.0 and 89.0%, respectively. The average relative abundance of *Proteobacteria* was higher in the HIV-bronchiectasis group than in CF 72.3% vs. 40.1%, respectively. In contrast, the average relative abundance of *Firmicutes* was higher within the CF group (49.0% vs. 22.2%). The remaining three phyla in decreasing order had an average relative abundance of: *Fusobacteria*, 2.4%; *Bacteroidetes*, 1.9% and *Actinobacteria*, 0.5% within the HIV-bronchiectasis group. For the CF groups *Bacteroidetes* 8.4%, *Fusobacteria* 1.0% and *Actinobacteria* 0.7% were the other predominant phyla. In addition, several samples were dominated by other phyla that contributed towards a significant proportion of the phyla assemblage. *Fusobacteria* was detected in HIV-bronchiectasis (BE) samples: BE6 (36.8%), BE12 (9.3%) and BE18 (6.7%), whereas *Bacteroidetes* were detected in BE15 (16.1%), BE18 (9.4%) and BE22 (12.9%). Five genera *Moryella*, *Parvimonas*, *Peptostreptococcus*, *Pseudomonas* and *Sneathia* were confined to HIV-bronchiectasis samples.

As with the HIV-bronchiectasis samples, the fluctuating dominance of *Proteobacteria* and *Firmicutes* was also observed within CF samples. *Proteobacteria* dominated two CF samples - CF1 and CF6 [69.7%, range 64.8 - 74.7%)], whereas *Firmicutes* dominated the remaining 4 CF samples [59.9%, range (47.1- 70.1%)]. In addition, *Bacteroidetes* were present at high relative abundances (range 4.0 - 20.8%) with the exception of sample CF1 in which the phylum was not detected. *Staphylococcus* was detected only within CF samples. Exacerbations had no impact on the microbial community composition.

Taxonomic affiliation at a genus level was used to explain the bacterial community profiles. *Haemophilus* had a higher prevalence in the HIV-bronchiectasis group (64.7%) than the CF group (28.0%). In contrast, *Streptococcus* was more prevalent in the CF group (41.4% vs. 15.2%) than in the HIV-bronchiectasis group. The genera assemblage harboured by each sample was structurally diverse. *Haemophilus* (*Proteobacteria* phylum) and *Streptococcus* (*Firmicutes* phylum) were the dominant genera within HIV-bronchiectasis and CF samples with combined average abundances of these two genera reaching 79.9 and 69.4% within each group, respectively.

**Fig. 5:**
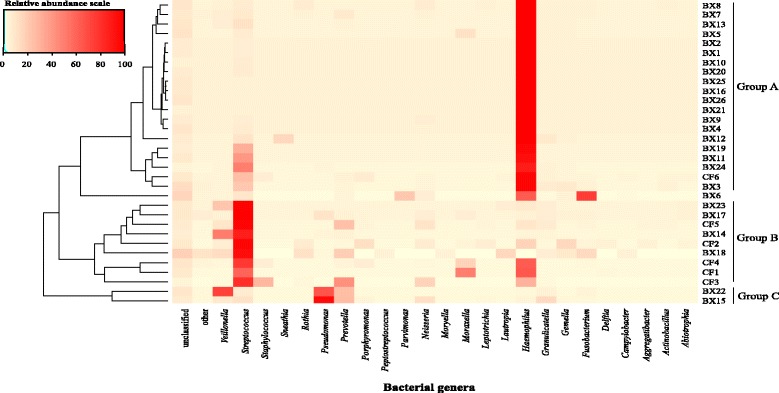
Heatmap showing the relationship between individual BX and CF samples and bacterial genera detected at a frequency abundance ≥1%. The UPGMA tree shown on the left side of the figure depicts hierarchical clustering of 26 BX and 5 CF samples based on Bray-Curtis dissimilarity coefficient

Compositional similarity within the genera assemblage of individual samples was displayed in the heat map, which was constructed following UPGMA hierarchical cluster analysis (Fig. 5). The samples were categorized into three distinct groupings that were distinguishable from one another based on their bacterial genera assemblage composition. The groups were designated as: Group A, *Haemophilus-*dominating with *Streptococcus*; Group B, *Streptococcus-*dominating with *Haemophilus* and Group C, *Pseudomonas-*dominating with *Prevotella*. The majority of the HIV-bronchiectasis samples and one CF samples (CF6) clustered within Group A, with relative abundances of *Haemophilus* 85.7% (range: 62.6 – 99.3%) and *Streptococcus* 27.3% (range: 0.1 – 34.6%). Group B included the CF samples, with the exception of CF6, as wells as 4 bronchiectasis samples (BE14, BE17, BE18, and BE23). This group was dominated by Streptococcus 53.8% (range: 32.8 – 79.2%), and *Haemophilus* 11.4% (range: 0.30- 35.4%). Group C contained only two samples (BE22 and BE15), which had high relative abundances of *Pseudomonas* (BE15 = 57.0% and BE22 = 35.0%) and *Prevotella* (BE15 = 14.8% and BE22 = 12.7%)*.* For lung function parameters there was no correlation between FEV_1_% nor FEF_25/75_% and the predominance of *Proteobacteria* (*r* = 0.154; *p* = 0. 0.4706 and *r* = 0.178; *p* = 0.4034), respectively.

## Discussion

In this study of the microbiome of children with HIV-associated bronchiectasis there was higher relative abundance of *Proteobacteria* when compared to a limited number of CF-bronchiectasis subjects, where *Furmicutes* predominated. *Pseudomonas* and *Prevotella* were also identified, but in less than 1% of the samples. There was no correlation between relative abundance of specific taxa and lung function parameters, although these children had significant morbidity with low lung functions. The community richness within the bronchiectasis subjects had relatively fewer OTUs and less sample heterogeneity when compared to the limited CF samples. Bacterial assemblage was not affected by the presence or absence of pulmonary exacerbations in the HIV-bronchiectasis group.

There is conflicting data in the literature with regards to the level of immunosuppression and its impact on the lung microbiome. In one study in HIV-positive adults with acute pneumonia in two cohorts in Uganda and San Francisco, the Ugandan subjects revealed a richer and more diverse microbiome and higher prevalence of *P. aeruginosa* despite having more advanced HIV-disease staging [24]. A more recent study has shown that HIV-infected subjects with advanced disease demonstrated decreased alpha diversity (richness and diversity) when compared to HIV-uninfected individuals and that this difference persisted up to 3 years after initiation of HAART [12]. These studies suggest that HIV may impact the interaction between host and environment via perturbation in the bacterial diversity in the respiratory tract. The question of the impact of innate immunity and HIV also requires further study; so far there is one study in HIV-positive children that demonstrated lower saliva bacterial species in the study group, despite comparable levels of secretory IgA to an uninfected cohort [25]. In HIV-positive individuals, the use of antimicrobials, antifungals and antiretroviral therapy may be contributing to the changing microbiome. The impact of polypharmacy and its role on dysbiosis in HIV still requires further elucidation. In the current study, we found lower bacterial diversity in the HIV-infected group when compared to an admittedly small control group of CF children.

Severity of lung disease has also been shown to impact the microbiome. In chronic obstructive pulmonary disease (COPD), more advance staging of disease with global initiative of chronic obstructive lung disease (GOLD) stage 4, was found to be associated with reduced bacterial diversity when compared to healthy individuals and COPD sufferers with milder disease [26, 27]. In the current study, the CF group had more preserved lung function than the HIV-bronchiectasis group and we postulate that the differences in severity of lung impairment may account for the differences in the microbiome in the two groups although the numbers were small. *Pseudomonas aeruginosa* was identified only in the HIV-bronchiectasis group, and this pathogen has been previously been associated with lung inflammation and reduced lung function [11, 28]. In the current study the  subjects with CF were younger and the sample size small, possibly explaining the lack of *P. aeruginosa* in this group.

Currently utilised tools for assessment of *P. aeruginosa* are crude, with bacterial densities, bacterial counts and bacterial numbers being unreliable to predict exacerbations [29–31]. Studies using the microbiome to guide therapeutic interventions have also yielded disappointing results. The use of antibiotics during exacerbation has been shown in both animal and human studies to have minimal impact on the microbial community composition, and the bacterial load with qPCR testing with the exception of *Pseudomonadales* [11, 29, 32]. The relative abundance of *Pseudomonas* as a target for assessment of treatment response is an attractive option, particularly in CF, bronchiectasis and COPD where *P. aeruginosa* colonization influences pulmonary outcomes and exacerbations. Further studies are needed in this area, particularly on the role of the microbial community and its change pre- and post-exacerbations; as well as for treatment response assessment.

The strength of the current study is that it provides pilot data on the microbiome in bronchiectasis in the context of HIV-infected children where little data exists. The differences shown reflects results found by other authors on the impact of HIV on the lung microbiome, showing reduced diversity and reduced richness [12, 24, 32]. There seems to be a signal of less diversity in HIV-bronchiectasis when compared to CF, although this should be interpreted with caution due to the small numbers in the CF group.

The study is limited by the small sample size and lack of an HIV-positive group without chronic lung disease, which could have provided insight to the effect of HIV-infection alone on the microbiome. Without the HIV “control” group, conclusions on the microbiome may not be based on lung disease severity but rather on the infection with HIV. A previous study by the Lung HIV Microbiome Project showed similarities in the microbiome of lower airway broncho-alveolar lavage samples of HIV-negative, HIV-positive HAART “naïve” and HIV-positive on HAART in adults [33]. In the current study, there was no comparison of the microbiome data with conventional sputum microscopy and sensitivity results. The number of CF ‘controls’ is also small and any conclusions should be interpreted with caution. We also collected induced samples and not broncho-alveolar protected brush samples, as previous studies in children have shown induced samples to provide adequate samples similar to those of the upper airway [34]. The numbers of patients with exacerbations are also small, limiting their interpretation and generalization.

The current findings, showing that *Haemophilus* and *Streptococcus* dominated the microbiome of both groups of patients were supported by previous culture based studies [6, 35]. Although the impact of reagent contamination on the microbiome was not addressed specifically, the possibility that these dominant groups could be directly linked to reagent contamination was small. *Haemophilus* was not identified as a typical contaminant previously and due to the high level of microbial biomass in all samples, high concentrations of DNA could be extracted [16, 36]. Comparison of the relative abundance data (Fig. 5) also did not provided any indication of issues with contamination of DNA in the reagents.

## Conclusion

The microbiome in children with HIV-associated bronchiectasis seems to be less rich, diverse and heterogeneous than in children with CF-bronchiectasis, with predominance of *Proteobacteria*.

## Abbreviations

BE: Bronchiectasis
CF: Cystic fibrosis
COPD: Chronic obstructive pulmonary disease
FEV1: Forced expiratory volume in one second
FVC: Forced vital capacity
GOLD: Global initiative of chronic obstructive lung disease
HAART: Highly active antiretroviral therapy
HIV: Human immunodeficiency virus infection
NMDS: Nonmetric multidimensional scaling
OTU: Operational taxonomy units
rRNA: Ribosomal ribonucleic acid
TB: Tuberculosis
WHO: World health organization
